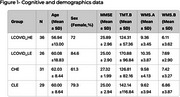# Long COVID and Low Education: Neuropsychological Findings from the Brazilian NeuroCovid Cohort

**DOI:** 10.1002/alz70857_105941

**Published:** 2025-12-24

**Authors:** Joana Emilia Senger, Luiza Santos Machado, Maiele Dornelles Silveira, João Pedro Ferrari‐Souza, Marco De Bastiani, Guilherme Povala, Wyllians Vendramini Borelli, Guilherme Bastos de Mello, João Pedro Uglione Da Ros, Arthur Viana Jotz, Matheus Fakhri Kadan, Graciane Radaelli, Tharick A Pascoal, Cristina Sebastião Matushita, Ricardo Benardi Soder, Artur Francisco Schumacher‐Schuh, Diogo O. Souza, Mychael V. Lourenco, Daniele de Paula Faria, Arthur Coutinho, Jaderson Costa da Costa, Débora Guerini de Souza, Eduardo R. Zimmer

**Affiliations:** ^1^ Universidade Federal do Rio Grande do Sul, Porto Alegre, RS, Brazil; ^2^ Department of Psychiatry and Neurochemistry, Institute of Neuroscience and Physiology, The Sahlgrenska Academy, University of Gothenburg, Gothenburg, VG, Sweden; ^3^ Universidade Federal Rio Grande do Sul, Brazil, Porto Alegre, RS, Brazil; ^4^ Universidade Federal do Rio Grande do Sul, Porto Alegre, Rio Grande do Sul, Brazil; ^5^ University of Pittsburgh, Pittsburgh, PA, USA; ^6^ Hospital Moinhos de Vento, Porto Alegre, Rio Grande do Sul, Brazil; ^7^ Pontifícia Universidade Católica do Rio Grande do Sul, Porto Alegre, Rio Grande do Sul, Brazil; ^8^ Universidade Luterana do Brazil, Canoas, RS, Brazil; ^9^ Pontifícia Universidade Católica do Rio Grande do Sul, Porto Alegre, RS, Brazil; ^10^ Federal University of Rio Grande do Sul, Porto Alegre, RS, Brazil; ^11^ Instituto do Cérebro do Rio Grande do Sul, Porto Alegre, RS, Brazil; ^12^ McGill University Research Centre for Studies in Aging, Montreal, QC, Canada; ^13^ University of Pittsburgh School of Medicine, Pittsburgh, PA, USA; ^14^ Departments of Psychiatry and Neurology, University of Pittsburgh School of Medicine, Pittsburgh, PA, USA; ^15^ Hospital de Clínicas de Porto Alegre, Porto Alegre, RS, Brazil; ^16^ UFRGS, Porto Alegre, Brazil; ^17^ Universidade Federal do Rio de Janeiro, Rio de Janeiro, RJ, Brazil; ^18^ Universidade de São Paulo, São Paulo, SP, Brazil; ^19^ University of São Paulo, São Paulo, São Paulo, Brazil; ^20^ Brain Institute of Rio Grande Do Sul, PUCRS, Porto Alegre, RS, Brazil; ^21^ McGill Centre for Studies in Agin, Montreal, QC, Canada

## Abstract

**Background:**

Long COVID is a chronic condition that persists for at least three months after SARS‐CoV‐2 infection. It is characterized by a wide range of symptoms, including neurological manifestations. This study aimed to investigate the influence of long COVID on neuropsychological performance in adults with different educational levels, categorized into high and low education groups.

**Method:**

We included community‐dwelling individuals from Porto Alegre, Brazil. Participants were divided into four groups based on education and long COVID status: Control‐Low Education (CLE), Control‐High Education (CHE), Long COVID‐Low Education (LCOVID_LE), and Long COVID‐High Education (LCOVID_HE). High education was defined as having more than 11 years of schooling. Neuropsychological assessments included the Mini‐Mental State Examination (MMSE), the Trail Making Test (TMT‐B), and the Wechsler Memory Scale (WMS‐R). Group differences in neuropsychological performance were analyzed using Analysis of Variance (ANOVA). Post hoc pairwise comparisons were conducted for significant main effects.a A Kruskal Wallis analysis was used for non‐parametric tests. Statistical analyses were performed using R software, with a significance threshold set at *p* <0.05p<0.05.

**Result:**

A total of 122 individuals were included, with a mean age of 59.6 years (± 14.9), of whom 73.8% were female. Demographic details are shown in Figure 1. Participants in the CLE group scored significantly lower on the MMSE than the CHE group (*p* = 0.007). Similarly, the LCOVID_LE group scored significantly lower than the CHE group (*p* = 0.009). A significant result was found between MMSE and education (*p* = 0.003) in Kruskal Wallis analysis. However, no significant effects of long COVID were observed in other neuropsychological domains assessed.

**Conclusion:**

Our study highlighted the significant interplay between educational attainment, which contributes to cognitive reserve, and the neurological manifestations of long COVID. Higher educational attainment may confer resilience against cognitive decline associated with long COVID. While long COVID was linked to lower global cognitive scores, no specific cognitive domain appeared particularly vulnerable. These findings underscore the importance of prioritizing support for vulnerable populations with low education to promote brain health.